# Redox Regulation of Protein Function via Cysteine S-Nitrosylation and Its Relevance to Neurodegenerative Diseases

**DOI:** 10.1155/2012/463756

**Published:** 2012-08-16

**Authors:** Mohd Waseem Akhtar, Carmen R. Sunico, Tomohiro Nakamura, Stuart A. Lipton

**Affiliations:** Del E. Webb Center for Neuroscience, Aging, and Stem Cell Research, Sanford-Burnham Medical Research Institute, 10901 North Torrey Pines Road, La Jolla, CA 92037, USA

## Abstract

Debilitating neurodegenerative diseases, such as Alzheimer's disease (AD) and Parkinson's disease (PD), can be attributed to neuronal cell damage in specific brain regions. An important hallmark of these diseases is increased oxidative and nitrosative stress that occurs via overproduction of highly reactive free radicals known as reactive oxygen species (ROS) and reactive nitrogen species (RNS). These molecules are normally removed by cellular antioxidant systems. Under physiological conditions, ROS/RNS are present at low levels, mediating several neurotrophic and neuroprotective signaling pathways. In contrast, under pathological conditions, there is a pronounced increase in ROS/RNS generation, impairing normal neurological function. Nitric oxide (NO) is one such molecule that functions as a signaling agent under physiological conditions but causes nitrosative stress under pathological conditions due to its enhanced production. As first reported by our group and colleagues, the toxic effects of NO can be in part attributed to thiol S-nitrosylation, a posttranslational modification of cysteine residues on specific proteins. Here, we review several reports appearing over the past decade showing that S-nitrosylation of an increasing number of proteins compromises important cellular functions, including mitochondrial dynamics, endoplasmic reticulum (ER) protein folding, and signal transduction, thereby promoting synaptic damage, cell death, and neurodegeneration.

## 1. Introduction

**Figure 1:**
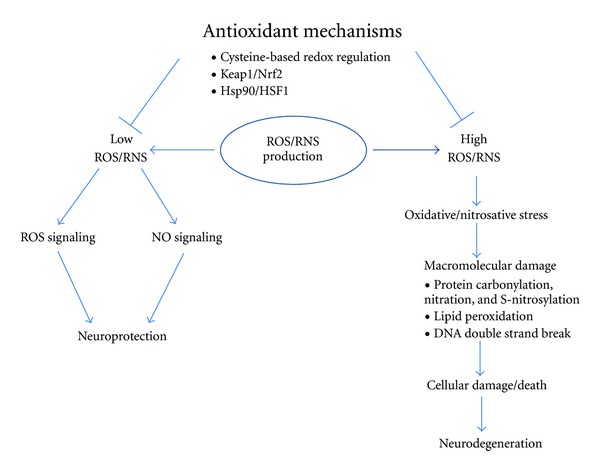
Imbalance in oxidant production and antioxidant mechanisms contributes to neurodegeneration. Under physiological conditions, antioxidant mechanisms such as cysteine-based redox regulation (Prx, Grx, Trx, glutathione (GSH), etc.), as well as transcriptional pathways represented by Keap1/Nrf2 and Hsp90/HSF1, maintain low concentrations of ROS/RNS in the neurons. These low levels of oxidants activate specific signaling pathways that subserve normal cell signaling and in fact may be neuroprotective in nature. On the other hand, under pathological situations, including AD and PD, there is a decrease in antioxidant mechanisms and increased oxidant production, effectively creating high levels of ROS/RNS. Oxidative/nitrosative stress generated in this manner can contribute to cell damage and results in neurodegeneration.

A delicate balance in redox state exists in cells, in large part because of production of ROS/RNS and the antioxidant systems that detoxify them. This homeostatic redox balance maintains a relatively low concentration of ROS/RNS. Under physiological conditions, ROS/RNS can activate specific signaling pathways required for diverse cellular functions, including cell growth and immune responses [1]. However, increased ROS/RNS production or decreased antioxidant capacity can lead to perturbation of the redox balance, causing oxidative/nitrosative stress [2] (Figure 1). We and others have demonstrated that sustained oxidative/nitrosative stress elicits counterattack mechanisms, including activation of transcriptional pathways that activate (i) endogenous antioxidant phase 2 enzymes (the Keap1/Nrf2 cascade) and (ii) chaperones for refolding misfolded proteins (heat-shock proteins of the Hsp90/HSF1 cascade). These transcription pathways can be activated directly by ROS/RNS or by electrophilic compounds generated in response to oxidation [3–6]. For example, upon reaction of an electrophile with Keap1, Nrf2 dissociates from the Keap1/Nrf2 complex in the cytoplasm and translocates into the nucleus to initiate transcription of phase 2 antioxidant genes [7–9]. HSF1 activates transcription of heat shock proteins to combat protein misfolding due to stress [10, 11]. If oxidant counteraction mechanisms, including activation of the Keap1/Nrf2 and Hsp90/HSF1 pathways, fail to combat ROS/RNS-related stress, cell injury, and death ensues (Figure 1). Synaptic loss and neuronal cell death due to excessive oxidative/nitrosative stress have been widely implicated in neurodegenerative disorders, including Alzheimer's disease (AD) and Parkinson's disease (PD).

ROS and RNS are highly reactive molecules or free radicals. For instance, free radical nitric oxide (NO) possesses an unpaired electron in its outer pi molecular orbital. Due to this nature, ROS and RNS can react somewhat indiscriminately with all classes of biological macromolecules (e.g., protein, lipid, DNA) and cause cellular damage (Figure 1). In this paper, we will specifically address the effect of nitrosative stress triggered by NO species that react to form protein S-nitrosothiols. It should be noted, however, that NO signaling can result in other types of posttranslational modifications, such as protein tyrosine nitration and S-glutathionylation, as well as reaction with heme, for example, to activate soluble guanylate cyclase to form cGMP [12].

## 2. Nitric Oxide Production and Signaling

Cellular production of NO from l-arginine is catalyzed by a family of enzymes known as NO synthases (NOSs). The NOS family consists of endothelial NOS (eNOS), neuronal NOS (nNOS), and inducible NOS (iNOS) [13], and all three NOS subtypes are expressed in the mammalian brain. For instance, Ca^2+^-dependent nNOS catalyzes production of NO predominantly in neurons, whereas Ca^2+^-independent iNOS is primarily (but not exclusively) involved in NO production within microglia and astrocytes [14].

**Figure 2:**
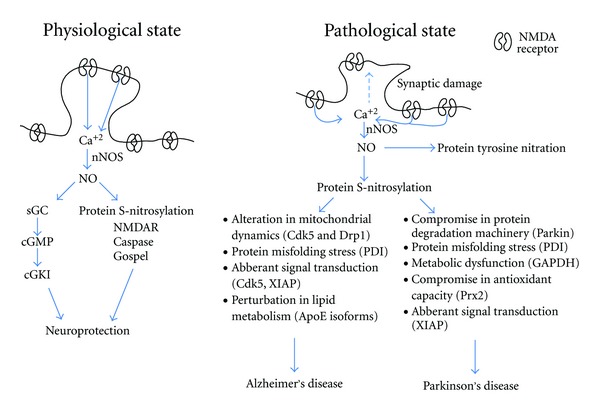
Nitric oxide signaling in neuroprotection and neurodegeneration. Synaptic activity results in NMDAR channel openings, allowing Ca^2+^ entry that can activate nNOS to generate NO in neurons. Under physiological conditions, low levels of NO are produced in neurons by synaptic activity to activate neuroprotective signaling pathways involving cGMP-CGKI or S-nitrosylation of several critical proteins (NMDARs, Gospel, etc.). Under pathological conditions, excessive Ca^2+^ enters primarily through extrasynaptic NMDARs, generating large concentrations of NO. The nitrosative stress thus generated contributes to synaptic damage and neuronal loss, in part by fostering aberrant protein S-nitrosylation. Various cellular processes, including mitochondrial dynamics, protein folding, lipid metabolism, protein degradation, and signal transduction pathways can be perturbed by aberrant protein S-nitrosylation. Compromise in one or several of these cell processes can contribute to neurodegeneration. It should be noted, however, that a number of additional pathways, not related to NO, that are triggered by synaptic activity can also contribute to neuroprotection, while a number of pathways affected by extrasynaptic NMDAR activity appear to be involved in neuronal cell injury and death [99].

Many excitatory synapses contain *N*-methyl-d-aspartate-type glutamate receptor- (NMDAR-) operated channels. Activation of these channels results in Ca^2+^ influx, triggering NO production by nNOS [15]. NO can undergo a number of reactions under normal physiological conditions. For example, NO reacts with soluble guanylate cyclase to produce cyclic GMP (cGMP) [16]. The second messenger cGMP then activates cyclic guanylate kinases (cGKs) [17, 18]. Once activated, cGKs can phosphorylate various physiological substrates in neurons, thereby controlling various important processes, including synaptic transmission and synaptic plasticity [14, 18] (Figure 2). An even more prominent physiological reaction of NO involves the posttranslational modification of S-nitrosylation or transfer of an NO group to a critical cysteine sulfhydryl to regulate protein function [19], in some sense analogous to phosphorylation of tyrosine, threonine, or serine residues. However, under pathological conditions, hyperactivation of NMDARs (often located at extrasynaptic or perisynaptic sites) causes massive Ca^2+^ influx and overproduction of NO [19–21]. Nitrosative stress due to NO overproduction compromises cellular signaling via aberrant protein S-nitrosylation and tyrosine nitration, which can contribute to neuronal cell injury or death [12] (Figure 2). In contrast, it should be noted that several of the proteins that are S-nitrosylated under physiological conditions, for example, the NMDAR itself and GOSPEL foster cell survival [22, 23].

## 3. Protein S-Nitrosylation

As alluded to above, protein S-nitrosylation is a reversible posttranslational modification whereby an NO group is covalently attached to a cysteine thiol group (or more properly, a thiolate anion, –S^−^) to form an S-nitroso derivative (R-SNO). Thus, we refer to S-nitrosylated proteins as SNO-proteins. Importantly, not all the cysteines in a protein can be S-nitrosylated. Cysteines that are surrounded by a particular amino-acid motif are likely candidates for this modification. This “SNO motif” is a consensus grouping of amino acids that consists of nucleophilic residues (generally an acid and a base), which may result from protein tertiary or even quaternary structure [24]. A specific modification by S-nitrosylation typically affects protein activity (either activating or inhibiting), thus mediating NO signaling pathways [23, 25, 26]. Proteins also can be denitrosylated (although transnitrosylation may also be involved) reportedly by redox-sensitive enzymes such as the thioredoxin (Trx) and S-nitroso-glutathione reductase systems, protein disulfide isomerase (PDI), and alcohol dehydrogenase (ADH) class III, which are now referred to as denitrosylases [27].

## 4. Implications of Protein S-Nitrosylation for Neurodegeneration

As discussed earlier, NO is produced in normal physiological conditions but does not induce nitrosative stress at low concentrations. However, under pathological neurodegenerative situations, NO production is highly increased, activating harmful signaling pathways, in large part due to aberrant protein S-nitrosylation. In this section, we will review several of the effects of protein S-nitrosylation in neurodegenerative diseases, including AD and PD.

### 4.1. Alzheimer's Disease

AD is one of, if not the most, common forms of dementia, resulting in progressive decline of intellectual and social abilities that cause problems in day-to-day life. AD is a neurodegenerative disorder as there is a tremendous amount of cell injury and loss in various parts of the brain, including the hippocampus and neocortex [28]. One of the important observations in AD pathogenesis is that synaptic deficits precede cellular death and correlate well with decline in intellectual function [29, 30]. Most AD cases (>95%) are sporadic, meaning that the majority of AD onset is not associated with obvious genetic mutations. Since AD mainly occurs in elderly people over 60 years old, age is one of the predisposing factors for the disease. One important hypothesis of aging and diseases of aging is the free radical theory, which states that an organism accumulates oxidative damage over time due to decreases in antioxidant systems and overproduction of free radicals [31, 32]. Consistent with this theory, several studies have clearly shown that AD brains exhibit increased oxidative/nitrosative stress [33]. We have found that several proteins critical to neuronal survival are S-nitrosylated and, in some cases, further oxidized in AD, thereby disrupting the normal activity of the protein and contributing to disease pathogenesis, as described below.

#### 4.1.1. S-Nitrosylation of Protein Disulfide Isomerase (PDI)

Once polypeptides are synthesized on ribosomes, those destined to be secreted are translocated to the endoplasmic reticulum (ER) for proper folding and disulfide bond formation. PDI is one of the enzymes that catalyze correct disulfide bond formation through a series of thiol-disulfide exchange reactions [34, 35]. In the absence of proper disulfide bond formation, proteins misfold and aggregate in the ER, resulting in ER stress [36]. If ER stress persists, it can result in cell death [37]. Several lines of research have implicated a role for ER stress in AD pathophysiology [38]. Our laboratory discovered that PDI is S-nitrosylated in human AD brain compared to control brain. S-nitrosylation of PDI facilitates further oxidation of cysteine residues to sulfenic (–SOH), sulfinic (–SO_2_H), and sulfonic (–SO_3_H) acid PDI derivatives. These redox modifications compromise PDI chaperone/protein folding function, leading to protein misfolding and ER stress [26]. These results highlighted the role of nitrosative stress and SNO-PDI in neuronal cell injury and death in AD.

#### 4.1.2. S-Nitrosylation of Dynamin Related Protein 1 (Drp1)

Neurons, and particularly their synaptic connections, require a tremendous amount of energy due to their high metabolic activity. Mitochondria, being the powerhouses of the cell, generate the vast majority of this energy. Recent reports suggest that to meet energy demand in an efficient manner, mitochondrial dynamics, consisting of fission and fusion events to generate new mitochondria, have to be carefully regulated [39]. Perturbation of mitochondrial dynamics can have deleterious effects on neuronal function and survival [40, 41]. Studies from our laboratory have shown that aberrant S-nitrosylation of Drp1 (a protein required for mitochondrial fission) hyperactivates Drp1, and, in turn, causes a dramatic increase in mitochondrial fission. We demonstrated that the altered mitochondrial dynamics due to S-nitrosylated Drp1 (SNO-Drp1) contributes to synaptic loss in neurons and subsequent neuronal cell death. In addition, SNO-Drp1 levels are significantly increased in postmortem sporadic human AD patient brains compared to controls [42]. Hence, this study clearly implicated the pathophysiological role of SNO-Drp1 in AD pathophysiology.

#### 4.1.3. Transnitrosylation of Cdk5 to Drp1

Molecular signaling pathways are central to cellular physiology and function. Several signaling molecules have been implicated in AD pathophysiology [43]. One such molecule is cyclin-dependent kinase 5 (Cdk5), the activity of which has been shown to be altered in AD [44]. Cdk5 in neurons does not function as a cell cycle regulator, yet it exerts control over various aspects of neuronal function including cell survival, neuronal migration, dendritic spine density, and synaptic plasticity [45–47]. In a recently published article from our laboratory, Qu et al. showed that Cdk5, in addition to being a kinase, is also a nitrosylase, capable of S-nitrosylating other targets involved in both AD and PD. Initially, we found that Cdk5 itself could be S-nitrosylated in an A*β*- and NMDAR-dependant manner in neurons due to generation of NO by these insults [25]. Furthermore, we showed that S-nitrosylation of Cdk5 results in activation and contributes to A*β*-induced dendritic spine loss, representing a decrease in synapses, the only pathological correlate to clinical dementia in AD. Moreover, SNO-Cdk5 levels are significantly increased in postmortem sporadic AD patient brains compared to age matched control brains. Importantly, SNO-Cdk5 then appears to contribute to synaptic failure by acting as an endogenous nitrosylase for Drp1, transferring the NO group from Cdk5 to Drp1 to form SNO-Drp1. This study revealed a role for protein S-nitrosylation of Cdk5 in aberrant cell signaling and links this nitrosylase activity to neuronal damage in AD [25].

#### 4.1.4. S-Nitrosylation of ApoE

Apolipoprotein E (ApoE) represents a major risk factor locus for late onset Alzheimer's disease [48]. The different isoforms of ApoE vary at their cysteine residues, which are potential sites for S-nitrosylation, as our group had observed a number of years ago. A recent study showed that all ApoE isoforms can bind nNOS and that ApoE2 and ApoE3 can be found in the S-nitrosylated state in human hippocampal lysates [49]. S-Nitrosylation of ApoE isoforms has been suggested to cause loss of binding to low density lipoprotein (LDL) receptors. Thus, S-nitrosylation of ApoE may affect lipid metabolism, which is postulated to affect the progression of AD.

The above-mentioned studies highlight some of the roles of S-nitrosylated proteins and how they can alter diverse cellular functions, including mitochondrial dynamics and synapse loss, ER protein folding, signal transduction pathways, and lipid metabolism, thereby affecting the progression of AD (Figure 2). In addition to these pathways, we suspect that there are many more pathways altered by protein S-nitrosylation in AD pathophysiology.

### 4.2. Parkinson's Disease

PD is second only to AD in the prevalence of neurodegenerative disorders. It affects approximately 1% of people over 65 years of age [50] and is characterized by motor sequencing impairment and often has a component of dementia. Although there are some symptomatic treatments for patients suffering from PD, currently there is no successful therapy to prevent progression or restore function. The histopathological aspects of PD include the loss of dopaminergic neurons, primarily in the substantia nigra *pars compacta*, often with the simultaneous presence of intracellular inclusions called Lewy bodies. Lewy bodies are mainly distributed in the substantia nigra, neocortex, basal forebrain nuclei, and hippocampus [51, 52]. Despite the fact that some familial cases have been identified, more than 95% of PD cases are reported as sporadic, some of which appear to be correlated with exposures to agricultural pesticides, herbicides, fungicides, heavy metals, or neurotoxins [53, 54], although this epidemiological information has remained contentious in some circles. We and others have shown that several of these environmental factors induce the generation of potentially toxic ROS/RNS species within neuronal cells [55]. Interestingly, dopaminergic neurons are especially vulnerable to oxidative/nitrosative stress, perhaps partly because of the oxidizing nature of dopamine. These observations have raised the hypothesis that in sporadic PD cases, oxidative or nitrosative stress contributes to PD pathogenesis via altering the function of PD-associated proteins. In several cases, the same gene product that is encoded in hereditary cases of PD may be affected by environmental factors to mimic the more rare genetic form or increase the susceptibility or severity of the hereditary phenotype, as highlighted below.

#### 4.2.1. S-Nitrosylation of Parkin

As an example, mutations in the parkin gene are known to cause many cases of the autosomal-recessive juvenile Parkinsonism and some rare cases of adult-onset PD [56–59]. The parkin gene encodes an ubiquitin E3 ligase that targets many proteins for proteasomal degradation and also has a neuroprotective role in PD-related apoptotic events [56, 60]. Mutations in the parkin gene result in the disturbances in parkin-mediated protein ubiquitination [61–63], which leads to the accumulation of potentially neurotoxic protein aggregates of parkin substrates with consequent dysfunction of the ubiquitin-proteasome system degradative pathway [61, 64, 65]. Interestingly, recent reports suggest that, independently from its ubiquitin-ligase role, parkin also functions as a transcriptional repressor of p53 to protect dopaminergic neurons from PD-related stress [66, 67].

In addition to these rare mutations, several environmental toxins that trigger oxidative/nitrosative stress are believed to affect the enzymatic activity of parkin protein. For instance, certain pesticides, herbicides, and fungicides that generate ROS and RNS and have been linked epidemiologically to PD can cause alterations in parkin solubility, inducing its aggregation and compromising its protective function. Parkin has multiple cysteine residues in its RING domain and elsewhere [66, 68], which can react with NO to form SNO-parkin. This S-nitrosylation reaction compromises parkin's neuroprotective function. Our group reported that S-nitrosylation of parkin initially increases E3 ligase activity, but with additional time this activity is inhibited. This dysfunctional E3 ligase activity is associated with abnormal protein aggregation resembling Lewy bodies, thus contributing to the parkinsonian phenotype [68]. Moreover, S-nitrosylation of parkin has also been found by our group and others in a mouse MPTP model of PD and in brains of human patients with Lewy body disease (LBD) and PD [68, 69]. These findings support our notion that posttranslational changes to PD-related proteins via S-nitrosylation or other oxidation reactions may well contribute to the etiology of sporadic PD.

#### 4.2.2. S-Nitrosylation of Peroxiredoxin

Our group and colleagues have also demonstrated that S-nitrosylation of another protein, peroxiredoxin 2 (Prx2), may be related to PD pathogenesis. Prxs are a highly abundant family of antioxidant enzymes that reduce intracellular peroxides by redox reactions [70–72]. Among the Prx enzymes, Prx2 is the most abundant in the mammalian brain and neurons. The active site cysteine residues in Prx2 reduce peroxides to H_2_O, thus forming a sulfenic acid (–SOH) derivative of Prx2. Subsequently, the oxidized Prx2 cysteine(s) can either form an intermolecular disulfide bond (–S–S–) with another Prx2 molecule, undergo reduction/regeneration back to free sulfhydryl (–SH) by thioredoxin (Trx), or be further oxidized (termed hyperoxidation) to produce a sulfinic (–SO_2_H) or sulfonic (–SO_3_H) acid derivative.

In several neurodegenerative diseases linked to oxidative/nitrosative stress, Prx2 levels are increased [73, 74], which may represent an attempt of the cell to counteract oxidative/nitrosative insult during neurodegeneration. We and others recently reported that Prx2 activity can be regulated *in vitro* and in cell-based systems by NO through S-nitrosylation of redox-active cysteine residues, which would prevent the reaction of this protein with peroxides, thus preventing the neuroprotective action of Prx2 [75, 76]. In human samples of PD brains and cell-based models of PD, S-nitrosylation of Prx2 has been found to be increased compared to control samples [75]. Since SNO-Prx2 cannot react with peroxide because the active cysteines are already nitrosylated, the normal redox cycle to detoxify ROS is disrupted, inducing oxidative stress that can contribute to neuronal cell death.

#### 4.2.3. S-Nitrosylation of XIAP

The protein X-linked inhibitor of apoptosis (XIAP) has also been found to be S-nitrosylated in several neurodegenerative disorders, including Alzheimer's, Parkinson's, and Huntington's diseases (HD), by our laboratory and others. Inhibitors of apoptosis (IAPs) are a family of proteins that regulate cell survival through binding to caspases to repress their catalytic activity [77, 78]. XIAP is the most commonly expressed and the most potent endogenous caspase inhibitor among the IAPs. XIAP has three copies of the baculovirus IAP repeat (BIR) domain and one RING domain at the C terminal. Biochemical and structural studies demonstrated that BIR domains confer the anticaspase activity [79], whereas the RING domain can act as an E3 ubiquitin ligase in the proteasome system [80–84].

Recent studies show a significant increase of S-nitrosylated XIAP in both cell-based and animal models of PD as well as in human brain samples from PD, AD, and HD patients [85, 86]. Our detailed experiments have identified that the RING domains of XIAP can react with NO by S-nitrosylation [85], although very high, nonphysiological concentrations of NO can also induce S-nitrosylation of the BIR domain [86]. S-Nitrosylation of XIAP at the RING domain inhibits its E3 ligase and antiapoptotic activity. Furthermore, we demonstrated recently that S-nitrosylated caspases can transfer their NO group to XIAP in a process called transnitrosylation. This reaction inhibits XIAP ubiquitin E3 ligase activity on caspases, thus effectively enhancing caspase activity and thus promoting proapoptotic signaling [85].

#### 4.2.4. S-Nitrosylation of GAPDH

Solomon Snyder's group has shown that the important metabolic enzyme glyceraldehyde-3-phosphate dehydrogenase (GAPDH) can be S-nitrosylated to form SNO-GAPDH. SNO-GAPDH manifests a loss of enzymatic activity [87]. More importantly, S-nitrosylated GAPDH efficiently binds Siah1 protein and then translocates to the nucleus. In the nucleus, this protein complex activates ubiquitination and thus degradation of several nuclear proteins, including nuclear receptor corepressor (N-COR); this process contributes to cell death [87]. These studies suggest that S-nitrosylation of GAPDH, on one hand, compromises its metabolic enzymatic activity, but, on the other hand, in conjunction with Siah1, forms an important signaling complex to promote cell death and neurodegeneration.

#### 4.2.5. S-Nitrosylation of PDI

As discussed above, protein misfolding and ER stress can be precipitated by S-nitrosylation of PDI, thereby potentially contributing to neuronal injury in AD. Our laboratory has also shown this scenario to be true in PD models. For example, when SH-SY5Y dopaminergic cells were treated with rotenone, a pesticide implicated in the pathogenesis of PD, we observed an increase in SNO-PDI levels concomitant with a decrease in PDI chaperone activity. Additionally, we found dramatically increased levels of SNO-PDI in human postmortem PD brains compared to controls [26]. Since ER stress due to protein misfolding is thought to contribute to the neurodegenerative process in PD [88], our finding of a substantial degree of SNO-PDI in PD brains has both pathogenic and therapeutic implications.

#### 4.2.6. S-Nitrosylation of DJ-1

Deletions or point mutations in the protein DJ-1 (PARK7) have been shown to be responsible for an early-onset, autosomal-recessive form of PD [89]. Interestingly, DJ-1-mediated signaling pathways have also been implicated in the much more common sporadic form of PD. It has been postulated that the increase in DJ-1 expression observed in cells undergoing nitrosative stress induced by the herbicide, paraquat, represents an attempt to protect the cells [90]. Consistent with this notion, DJ-1 knockdown makes neuronal-like cells more susceptible to peroxide-, MPP^+^-, and 6-hydroxydopamine-induced cell death [91, 92]. Additionally, DJ-1 deficient flies [93–96] or mice [97] are more vulnerable to environmental neurotoxins associated with dopaminergic degeneration. Sequence analysis of protein DJ-1 reveals three potentially redox-active cysteine residues, two of which (Cys46 and Cys53) appear to be susceptible to S-nitrosylation *in vitro* and in cell-based systems [98]. Our group has also observed S-nitrosylation of a critical redox-active cysteine in the crystal structure of DJ-1. These findings and others suggest that posttranslational modifications of DJ-1, including protein S-nitrosylation, can disrupt the antioxidant action of DJ-1 in dopaminergic neurons, rendering them more susceptible to damage in sporadic PD. However, the elucidation of additional effects of SNO-DJ-1 in PD will require further investigation.

In summary, a number of studies suggest that nitrosative stress contributes to PD pathogenesis by altering neuroprotective proteins such as parkin, Prx2, PDI, GAPDH, and XIAP (Figure 2). These findings indicate that aberrant S-nitrosylation reactions may play an important role in this neurodegenerative disorder, providing additional insight into nitrosative mechanisms of PD pathogenesis as well as potential novel targets for the treatment of PD.

## 5. Conclusions

Nitric oxide signaling can be both beneficial and harmful to the nervous system depending on (i) the concentration of NO and (ii) the cell signaling pathways affected by various levels of NO. Physiological levels of NO activate both cGMP-cGKI and S-nitrosylation pathways responsible for various physiological processes, including those affecting synaptic transmission and plasticity. In contrast, high levels of NO compromise cellular functions by a variety of posttranslational modifications including aberrant S-nitrosylation reactions that would not normally occur in the presence of physiological levels of NO. In this paper, we have discussed data accumulated over the past several years that highlight the importance of protein S-nitrosylation in perturbing vital cell functions, including mitochondrial dynamics, protein folding, ubiquitination, synaptic transmission, and signal transduction pathways. Alteration of one or several of these events contributes to neuronal cell death and the development of neurodegenerative disorders (Figure 2). Although we have discussed the role of S-nitrosylation of several proteins here, including Drp1, PDI, GAPDH, ApoE, parkin, XIAP, Prx2, and DJ-1 in AD and PD, this list is by no means complete. Proteome-wide studies have already found hundreds, if not thousands, of proteins that are S-nitrosylated [99]. Future studies will unravel the role of S-nitrosylation of additional proteins in various cellular cascades and its implications for the pathogenesis and treatment of neurodegenerative disorders.

## References

[1] Finkel T (2011). Signal transduction by reactive oxygen species. *Journal of Cell Biology*.

[2] Imlay JA (2003). Pathways of oxidative damage. *Annual Review of Microbiology*.

[3] Fourquet S, Guerois R, Biard D, Toledano MB (2010). Activation of NRF2 by nitrosative agents and H_2_O_2_ involves KEAP1 disulfide formation. *Journal of Biological Chemistry*.

[4] Groeger AL, Freeman BA (2010). Signaling actions of electrophiles: anti-inflammatory therapeutic candidates. *Molecular Interventions*.

[5] Akaike T, Fujii S, Sawa T, Ihara H (2010). Cell signaling mediated by nitrated cyclic guanine nucleotide. *Nitric Oxide*.

[6] Satoh T, Lipton SA (2007). Redox regulation of neuronal survival mediated by electrophilic compounds. *Trends in Neurosciences*.

[7] Satoh T, Okamoto SI, Cui J (2006). Activation of the Keap1/Nrf2 pathway for neuroprotection by electrophillic phase II inducers. *Proceedings of the National Academy of Sciences of the United States of America*.

[8] Satoh T, Kosaka K, Itoh K (2008). Carnosic acid, a catechol-type electrophilic compound, protects neurons both in vitro and in vivo through activation of the Keap1/Nrf2 pathway via S-alkylation of targeted cysteines on Keap1. *Journal of Neurochemistry*.

[9] Kraft AD, Johnson DA, Johnson JA (2004). Nuclear factor E2-related factor 2-dependent antioxidant response element activation by tert-butylhydroquinone and sulforaphane occurring preferentially in astrocytes conditions neurons against oxidative insult. *Journal of Neuroscience*.

[10] Morimoto RI (2008). Proteotoxic stress and inducible chaperone networks in neurodegenerative disease and aging. *Genes and Development*.

[11] Bukau B, Weissman J, Horwich A (2006). Molecular chaperones and protein quality control. *Cell*.

[12] Martínez-Ruiz A, Cadenas S, Lamas S (2011). Nitric oxide signaling: classical, less classical, and nonclassical mechanisms. *Free Radical Biology and Medicine*.

[13] Forstermann U, Schmidt HHHW, Pollock JS (1991). Isoforms of nitric oxide synthase. Characterization and purification from different cell types. *Biochemical Pharmacology*.

[14] Steinert JR, Chernova T, Forsythe ID (2010). Nitric oxide signaling in brain function, dysfunction, and dementia. *Neuroscientist*.

[15] Sattler R, Xiong Z, Lu WY, Hafner M, MacDonald JF, Tymianski M (1999). Specific coupling of NMDA receptor activation to nitric oxide neurotoxicity by PSD-95 protein. *Science*.

[16] Russwurm M, Koesling D (2004). NO activation of guanylyl cyclase. *EMBO Journal*.

[17] Hofmann F (2005). The biology of cyclic GMP-dependent protein kinases. *Journal of Biological Chemistry*.

[18] Francis SH, Busch JL, Corbin JD (2010). cGMP-dependent protein kinases and cGMP phosphodiesterases in nitric oxide and cGMP action. *Pharmacological Reviews*.

[19] Lipton SA, Choi YB, Pan ZH (1993). A redox-based mechanism for the neuroprotective and neurodestructive effects of nitric oxide and related nitroso-compounds. *Nature*.

[20] Dawson VL, Dawson TM, London ED, Bredt DS, Snyder SH (1991). Nitric oxide mediates glutamate neurotoxicity in primary cortical cultures. *Proceedings of the National Academy of Sciences of the United States of America*.

[21] Bonfoco E, Krainc D, Ankarcrona M, Nicotera P, Lipton SA (1995). Apoptosis and necrosis: two distinct events induced, respectively, by mild and intense insults with N-methyl-D-aspartate or nitric oxide/superoxide in cortical cell cultures. *Proceedings of the National Academy of Sciences of the United States of America*.

[22] Sen N, Hara MR, Ahmad AS (2009). GOSPEL: a neuroprotective protein that binds to GAPDH upon S-nitrosylation. *Neuron*.

[23] Choi YB, Tenneti L, Le DA (2000). Molecular basis of NMDA receptor-coupled ion channel modulation by S- nitrosylation. *Nature Neuroscience*.

[24] Hess DT, Matsumoto A, Kim SO, Marshall HE, Stamler JS (2005). Protein S-nitrosylation: purview and parameters. *Nature Reviews Molecular Cell Biology*.

[25] Qu J, Nakamura T, Cao G (2011). S-Nitrosylation activates Cdk5 and contributes to synaptic spine loss induced by beta-amyloid peptide. *Proceedings of the National Academy of Sciences of the United States of America*.

[26] Uehara T, Nakamura T, Yao D (2006). S-Nitrosylated protein-disulphide isomerase links protein misfolding to neurodegeneration. *Nature*.

[27] Benhar M, Forrester MT, Stamler JS (2009). Protein denitrosylation: enzymatic mechanisms and cellular functions. *Nature Reviews Molecular Cell Biology*.

[28] LaFerla FM, Green KN, Oddo S (2007). Intracellular amyloid-*β* in Alzheimer’s disease. *Nature Reviews Neuroscience*.

[29] Selkoe DJ (2002). Alzheimer’s disease is a synaptic failure. *Science*.

[30] Terry RD, Masliah E, Salmon DP (1991). Physical basis of cognitive alterations in Alzheimer’s disease: synapse loss is the major correlate of cognitive impairment. *Annals of Neurology*.

[31] Harman D (1956). Aging: a theory based on free radical and radiation chemistry. *Journal of Gerontology*.

[32] Beckman KB, Ames BN (1998). The free radical theory of aging matures. *Physiological Reviews*.

[33] Sayre LM, Perry G, Smith MA (2008). Oxidative stress and neurotoxicity. *Chemical Research in Toxicology*.

[34] Lyles MM, Gilbert HF (1991). Catalysis of the oxidative folding of ribonuclease A by protein disulfide isomerase: pre-steady-state kinetics and the utilization of the oxidizing equivalents of the isomerase. *Biochemistry*.

[35] Lyles MM, Gilbert HF (1991). Catalysis of the oxidative folding of ribonuclease A by protein disulfide isomerase: dependence of the rate on the composition of the redox buffer. *Biochemistry*.

[36] Kaufman RJ (1999). Stress signaling from the lumen of the endoplasmic reticulum: coordination of gene transcriptional and translational controls. *Genes and Development*.

[37] Scheper W, Hoozemans JJM (2009). Endoplasmic reticulum protein quality control in neurodegenerative disease: the good, the bad and the therapy. *Current Medicinal Chemistry*.

[38] Doyle KM, Kennedy D, Gorman AM (2011). Unfolded proteins and endoplasmic reticulum stress in neurodegenerative disorders. *Journal of Cellular and Molecular Medicine*.

[39] Chen H, Chan DC (2009). Mitochondrial dynamics—fusion, fission, movement, and mitophagy—in neurodegenerative diseases. *Human Molecular Genetics*.

[40] Detmer SA, Chan DC (2007). Functions and dysfunctions of mitochondrial dynamics. *Nature Reviews Molecular Cell Biology*.

[41] Okamoto K, Shaw JM (2005). Mitochondrial morphology and dynamics in yeast and multicellular eukaryotes. *Annual Review of Genetics*.

[42] Cho DH, Nakamura T, Fang J (2009). *β*-Amyloid-related mitochondrial fission and neuronal injury. *Science*.

[43] Frautschy SA, Cole GM (2010). Why pleiotropic interventions are needed for alzheimer’s disease. *Molecular Neurobiology*.

[44] Patrick GN, Zukerberg L, Nikolic M, De La Monte S, Dikkes P, Tsai LH (1999). Conversion of p35 to p25 deregulates Cdk5 activity and promotes neurodegeneration. *Nature*.

[45] Ohshima T, Ward JM, Huh CG (1996). Targeted disruption of the cyclin-dependent kinase 5 gene results in abnormal corticogenesis, neuronal pathology and perinatal death. *Proceedings of the National Academy of Sciences of the United States of America*.

[46] Xie Z, Sanada K, Samuels BA, Shih H, Tsai LH (2003). Serine 732 phosphorylation of FAK by Cdk5 is important for microtubule organization, nuclear movement, and neuronal migration. *Cell*.

[47] Kim Y, Sung JY, Ceglia I (2006). Phosphorylation of WAVE1 regulates actin polymerization and dendritic spine morphology. *Nature*.

[48] Bertram L, Lill CM, Tanzi RE (2010). The genetics of alzheimer disease: back to the future. *Neuron*.

[49] Abrams AJ, Farooq A, Wang G (2011). S-nitrosylation of ApoE in Alzheimer’s disease. *Biochemistry*.

[50] Mayeux R, Marder K, Cote LJ (1988–1993). The frequency of idiopathic Parkinson's disease by age, ethnic group, and sex in northern Manhattan. *American Journal of Epidemiology*.

[51] Giasson BI, Lee VMY (2003). Are ubiquitination pathways central to Parkinson’s disease?. *Cell*.

[52] Jenner P (2003). Oxidative stress in Parkinson's disease. *Annals of Neurology*.

[53] Betarbet R, Sherer TB, MacKenzie G, Garcia-Osuna M, Panov AV, Greenamyre JT (2000). Chronic systemic pesticide exposure reproduces features of Parkinson’s disease. *Nature Neuroscience*.

[54] Langston JW (2002). Parkinson’s disease: current and future challenges. *NeuroToxicology*.

[55] Miller RL, James-Kracke M, Sun GY, Sun AY (2009). Oxidative and inflammatory pathways in parkinson’s disease. *Neurochemical Research*.

[56] Kitada T, Asakawa S, Hattori N (1998). Mutations in the parkin gene cause autosomal recessive juvenile parkinsonism. *Nature*.

[57] Lucking CB, Durr A, Bonifati V (2000). Association between early-onset Parkinson's disease and mutations in the parkin gene. *The New England Journal of Medicine*.

[58] Oliveira SA, Scott WK, Martin ER (2003). Parkin mutations and susceptibility alleles in late-onset Parkinson's disease. *Annals of Neurology*.

[59] Shimura H, Hattori N, Kubo SI (2000). Familial Parkinson disease gene product, parkin, is a ubiquitin-protein ligase. *Nature Genetics*.

[60] Jiang H, Ren Y, Zhao J, Feng J (2004). Parkin protects human dopaminergic neuroblastoma cells against dopamine-induced apoptosis. *Human Molecular Genetics*.

[61] Dawson TM, Dawson VL (2003). Molecular pathways of neurodegeneration in Parkinson’s disease. *Science*.

[62] Feany MB, Pallanck LJ (2003). Parkin: a multipurpose neuroprotective agent?. *Neuron*.

[63] Von Coelln R, Dawson VL, Dawson TM (2004). Parkin-associated Parkinson’s disease. *Cell and Tissue Research*.

[64] Bence NF, Sampat RM, Kopito RR (2001). Impairment of the ubiquitin-proteasome system by protein aggregation. *Science*.

[65] Masliah E, Rockenstein E, Veinbergs I (2000). Dopaminergic loss and inclusion body formation in *α*-synuclein mice: implications for neurodegenerative disorders. *Science*.

[66] da Costa CA, Sunyach C, Giaime E (2009). Transcriptional repression of p53 by parkin and impairment by mutations associated with autosomal recessive juvenile Parkinson’s disease. *Nature Cell Biology*.

[67] Alves da Costa C, Checler F (2011). Apoptosis in Parkinson’s disease: is p53 the missing link between genetic and sporadic Parkinsonism?. *Cellular Signalling*.

[68] Yao D, Gu Z, Nakamura T (2004). Nitrosative stress linked to sporadic Parkinson’s disease: S-nitrosylation of parkin regulates its E3 ubiquitin ligase activity. *Proceedings of the National Academy of Sciences of the United States of America*.

[69] Chung KKK, Thomas B, Li X (2004). S-nitrosylation of parkin regulates ubiquitination and compromises parkin’s protective function. *Science*.

[70] Sue GR, Ho ZC, Kim K (2005). Peroxiredoxins: a historical overview and speculative preview of novel mechanisms and emerging concepts in cell signaling. *Free Radical Biology and Medicine*.

[71] Rhee SG, Kang SW, Jeong W, Chang TS, Yang KS, Woo HA (2005). Intracellular messenger function of hydrogen peroxide and its regulation by peroxiredoxins. *Current Opinion in Cell Biology*.

[72] Wood ZA, Schröder E, Harris JR, Poole LB (2003). Structure, mechanism and regulation of peroxiredoxins. *Trends in Biochemical Sciences*.

[73] Kim SH, Fountoulakis M, Cairns N, Lubec G (2001). Protein levels of human peroxiredoxin subtypes in brains of patients with Alzheimer’s disease and Down Syndrome. *Journal of Neural Transmission, Supplement*.

[74] Krapfenbauer K, Engidawork E, Cairns N, Fountoulakis M, Lubec G (2003). Aberrant expression of peroxiredoxin subtypes in neurodegenerative disorders. *Brain Research*.

[75] Fang J, Nakamura T, Cho DH, Gu Z, Lipton SA (2007). S-nitrosylation of peroxiredoxin 2 promotes oxidative stress-induced neuronal cell death in Parkinson’s disease. *Proceedings of the National Academy of Sciences of the United States of America*.

[76] Romero-Puertas MC, Laxa M, Mattè A (2007). S-nitrosylation of peroxiredoxin II E promotes peroxynitrite-mediated tyrosine nitration. *Plant Cell*.

[77] Eckelman BP, Salvesen GS, Scott FL (2006). Human inhibitor of apoptosis proteins: why XIAP is the black sheep of the family. *EMBO Reports*.

[78] Salvesen GS, Duckett CS (2002). IAP proteins: blocking the road to death’s door. *Nature Reviews Molecular Cell Biology*.

[79] Fuentes-Prior P, Salvesen GS (2004). The protein structures that shape caspase activity, specificity, activation and inhibition. *Biochemical Journal*.

[80] MacFarlane M, Merrison W, Bratton SB, Cohen GM (2002). Proteasome-mediated degradation of Smac during apoptosis: XIAP promotes Smac ubiquitination in vitro. *Journal of Biological Chemistry*.

[81] Schile AJ, García-Fernández M, Steller H (2008). Regulation of apoptosis by XIAP ubiquitin-ligase activity. *Genes and Development*.

[82] Suzuki Y, Nakabayashi Y, Takahashi R (2001). Ubiquitin-protein ligase activity of X-linked inhibitor of apoptosis protein promotes proteasomal degradation of caspase-3 and enhances its anti-apoptotic effect in Fas-induced cell death. *Proceedings of the National Academy of Sciences of the United States of America*.

[83] Vaux DL, Silke J (2005). IAPs, RINGs and ubiquitylation. *Nature Reviews Molecular Cell Biology*.

[84] Yang Y, Fang S, Jensen JP, Weissman AM, Ashwell JD (2000). Ubiquitin protein ligase activity of IAPs and their degradation in proteasomes in response to apoptotic stimuli. *Science*.

[85] Nakamura T, Wang L, Wong CCL (2010). Transnitrosylation of XIAP regulates caspase-dependent neuronal cell death. *Molecular Cell*.

[86] Tsang AHK, Lee YIL, Ko HS (2009). S-nitrosylation of XIAP compromises neuronal survival in Parkinson’s disease. *Proceedings of the National Academy of Sciences of the United States of America*.

[87] Hara MR, Agrawal N, Kim SF (2005). S-nitrosylated GAPDH initiates apoptotic cell death by nuclear translocation following Siah1 binding. *Nature Cell Biology*.

[88] Wang HQ, Takahashi R (2007). Expanding insights on the involvement of endoplasmic reticulum stress in Parkinson’s disease. *Antioxidants and Redox Signaling*.

[89] Bonifati V, Rizzu P, Van Baren MJ (2003). Mutations in the DJ-1 gene associated with autosomal recessive early-onset parkinsonism. *Science*.

[90] Mitsumoto A, Nakagawa Y, Takeuchi A, Okawa K, Iwamatsu A, Takanezawa Y (2001). Oxidized forms of peroxiredoxins and DJ-1 on two-dimensional gels increased in response to sublethal levels of paraquat. *Free Radical Research*.

[91] Taira T, Saito Y, Niki T, Iguchi-Ariga SMM, Takahashi K, Ariga H (2004). DJ-1 has a role in antioxidative stress to prevent cell death. *EMBO Reports*.

[92] Yokota T, Sugawara K, Ito K, Takahashi R, Ariga H, Mizusawa H (2003). Down regulation of DJ-1 enhances cell death by oxidative stress, ER stress, and proteasome inhibition. *Biochemical and Biophysical Research Communications*.

[93] Meulener M, Whitworth AJ, Armstrong-Gold CE (2005). Drosophila DJ-1 mutants are selectively sensitive to environmental toxins associated with Parkinson’s disease. *Current Biology*.

[94] Menzies FM, Yenisetti SC, Min KT (2005). Roles of Drosophila DJ-1 in survival of dopaminergic neurons and oxidative stress. *Current Biology*.

[95] Yang Y, Gehrke S, Haque ME (2005). Inactivation of Drosophila DJ-1 leads to impairments of oxidative stress response and phosphatidylinositol 3-kinase/Akt signaling. *Proceedings of the National Academy of Sciences of the United States of America*.

[96] Park J, Sung YK, Cha GH, Sung BL, Kim S, Chung J (2005). Drosophila DJ-1 mutants show oxidative stress-sensitive locomotive dysfunction. *Gene*.

[97] Kim RH, Smith PD, Aleyasin H (2005). Hypersensitivity of DJ-1-deficient mice to 1-methyl-4-phenyl-1,2,3,6- tetrahydropyrindine (MPTP) and oxidative stress. *Proceedings of the National Academy of Sciences of the United States of America*.

[98] Ito G, Ariga H, Nakagawa Y, Iwatsubo T (2006). Roles of distinct cysteine residues in S-nitrosylation and dimerization of DJ-1. *Biochemical and Biophysical Research Communications*.

[99] Seth D, Stamler JS (2011). The SNO-proteome: causation and classifications. *Current Opinion in Chemical Biology*.

